# Bacterial community dynamics during the early stages of biofilm formation in a chlorinated experimental drinking water distribution system: implications for drinking water discolouration

**DOI:** 10.1111/jam.12516

**Published:** 2014-04-29

**Authors:** I Douterelo, R Sharpe, J Boxall

**Affiliations:** 1 Pennine Water Group, Department of Civil and Structural Engineering, University of SheffieldSheffield, UK; 2 School of Civil and Building Engineering, Loughborough UniversityLoughborough, UK

**Keywords:** 16s rRNA sequencing, bacterial community structure, biofilm development, discolouration, drinking water distribution systems, terminal restriction fragment length polymorphism

## Abstract

**Aims:**

To characterize bacterial communities during the early stages of biofilm formation and their role in water discolouration in a fully representative, chlorinated, experimental drinking water distribution systems (DWDS).

**Methods and Results:**

Biofilm development was monitored in an experimental DWDS over 28 days; subsequently the system was disturbed by raising hydraulic conditions to simulate pipe burst, cleaning or other system conditions. Biofilm cell cover was monitored by fluorescent microscopy and a fingerprinting technique used to assess changes in bacterial community. Selected samples were analysed by cloning and sequencing of the 16S rRNA gene. Fingerprinting analysis revealed significant changes in the bacterial community structure over time (*P* < 0·05). Cell coverage increased over time accompanied by an increase in bacterial richness and diversity.

**Conclusions:**

Shifts in the bacterial community structure were observed along with an increase in cell coverage, bacterial richness and diversity. Species related to *Pseudomonas* spp. and *Janthinobacterium* spp. dominated the process of initial attachment. Based on fingerprinting results, the hydraulic regimes did not affect the bacteriological composition of biofilms, but they did influence their mechanical stability.

**Significance and Importance of the Study:**

This study gives a better insight into the early stages of biofilm formation in DWDS and will contribute to the improvement of management strategies to control the formation of biofilms and the risk of discolouration.

## Introduction

It is commonly accepted that the use of a disinfectant residual such as chlorine in drinking water distribution systems (DWDS) does not completely prevent bacterial occurrence. Free-living bacteria can enter the distribution system through, for example, the treatment works, cross-connections or contamination ingress and can adhere to the pipe inner surfaces and form biofilms (Szewzyk *et al.* 2000). Biofilms are an advantageous way of living in hostile environments (Costerton *et al.* 1987; Simoes *et al.* 2010), and most of micro-organisms present in DWDS are able to survive by forming biofilms (Flemming *et al.* 2002; Batté *et al.* 2003). The process of biofilm formation on surfaces can be relatively fast even in chlorinated networks. Morvay *et al*. (2011) reported that biofilm formation on different plumbing material in chlorinated drinking water systems reaches values of 10^7 ^cells cm^−2^ after only 30 days.

Biofilms are structurally complex and consist of micro-organisms attached to a surface and to each other and embedded in an extracellular polymeric matrix (EPS) made of polysaccharides, proteins, extracellular DNA, etc. (Lopes *et al.* 2009). The EPS of biofilms offers protection to the direct action of disinfectants and also provides physical stability against the influence of shear forces (Flemming and Wingender 2010). Even in highly oligotrophic environments, such as DWDS, biofilms are diverse microbial ecosystems where different micro-organisms can coexist interacting with each other, acting as a unique entity and contributing with their different metabolic capabilities to the acquisition of nutrients (Stoodley *et al.* 2002). Microbial biofilms can modify the quality of drinking water both due to their presence and through their metabolic activities. The growth and accumulation of biofilms can modify hydraulics within the pipes, including pipe clogging, enhanced corrosion in metallic pipes and changes in water taste and odour (Zacheus *et al.* 2000). If the hydraulic conditions change in a way that overcomes biofilm adhesive forces, biofilms can detach from the pipe surfaces and contribute to the deterioration of water quality including discolouration (Ginige *et al.* 2011) and even have the potential to release pathogens into the bulk water.

**Figure 1:**
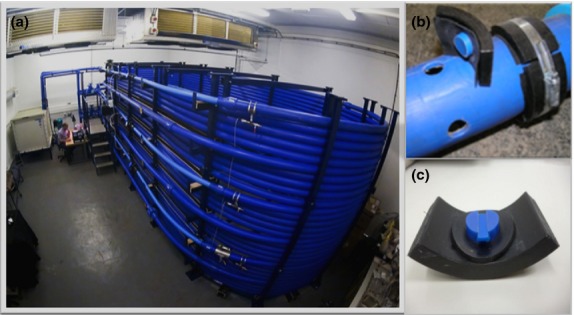
(a) full-scale laboratory pipe loop experimental facility at the University of Sheffield, (b) section of pipe designed to fit PWG coupons and (c) PWG coupon showing the insert and the outer part of the coupon.

It is generally accepted that DWDS are inhabited by different bacterial species that can form biofilms (Simoes *et al.* 2007b; Li *et al.* 2010). Using a model water distribution system with stainless steel plugs and operated under turbulent flow, Martiny *et al*. (2003) demonstrated that biofilm development is a dynamic process where bacterial succession occurs. However, most biofilm studies in aquatic environments are carried out under idealized laboratory conditions, using bench-top reactors and/or inoculating a limited number of micro-organisms (Moritz *et al.* 2010; Yu *et al.* 2010). As a consequence, little is known about which bacteria are involved in the initial development of biofilms under realistic conditions in water distribution networks. In this study, we use a full-scale experimental DWDS (Fig.1) which fully recreates hydraulic and physico-chemical conditions of real distribution systems to obtain more accurate information about the initial process of biofilm formation. The development of this test loop facility offers a significant advance in overcoming the difficulties of bench-scale experimentation. The facility was operated to allow a material accumulation phase, to represent material development in the network, as well as a flushing/mobilisation phase, to represent a network disturbance with the potential to cause biofilm mobilisation and associated discolouration.

In general, the formation of a biofilm is a successional process that can take years (Martiny *et al.* 2003) but starts when free-living bacteria (i.e. planktonic) attach to surfaces under certain conditions. These primary colonizing micro-organisms, mainly bacteria, start growing and are able to modify the substratum, providing more adhesion sites, and allowing for the colonization by other micro-organisms (Lee *et al.* 2008; Andrews *et al.* 2010). Different factors can affect the initial adhesion of planktonic cells to surfaces. In DWDS, these are likely to include pipe characteristics (material, diameter and roughness), source water parameters (e.g. pH, temperature and organic matter content), hydrodynamic conditions (flow, shear stress, etc.) and the characteristics of the bacterial cells themselves such as ability to produce extracellular polymeric substance, cell hydrophobicity and motility (Liu *et al.* 2004; Simoes *et al.* 2007a).

In this study, we explore who are the primary colonizers of a chlorinated distribution system, which bacteria are able to leave the bulk water and adhere to the pipe surfaces, how they are changing in their first month of biofilm formation and whether different hydraulic regimes affected their ability for initial adherence and the physical structure of the biofilm itself.

## Material and methods

### Experimental drinking water distribution system

To achieve the aims of this article, experiments were conducted in a purpose-built, temperature-controlled re-circulating test loop facility (Fig.1). The temperature-controlled test facility has been previously described (Douterelo *et al.* 2013). Briefly, the system has a total volume of 4·5 m^3^ and consists of three 200-m-long high-density polyethylene (HDPE) recirculating loops, fed by a common pump and returning to a common closed reservoir. The system was fitted with a trickle feed from the local water network and is set to obtain a system residence time of 24 h. The temperature for the experiment reported here was set at 16°C to reproduce average UK temperatures in DWDS during warmer months. The flow in each loop was individually controlled to generate different hydraulic regimes. The hydraulic regimes were: steady-state conditions (0·4 l s^−1^) and two variable flows with different daily hydraulic patterns based on costumer's drinking water demand in the UK; low varied flow, ranging from 0·2 to 0·5 l s^−1^ and highly varied flow ranging from 0·2 to 0·8 l s^−1^ (Husband *et al.* 2008).

Before starting the experiment, the test facility was disinfected with 20 mg l^−1^ of RODOLITE H (RODOL Ltd, Liverpool, UK) by flushing the system for three turnovers at the maximum flow rate (4·2 l s^−1^) and then being left stagnant with RODOLITE for 24 h. After that period, the system was flushed again at the maximum flow rate with fresh water until the levels of chlorine were similar to those of the local tap water. After disinfection of the facility, sterile PWG coupons of the same material (Deines *et al.* 2010) were fitted along and around the pipe length of each loop. Coupons were removed for biofilm analysis at days 0, 3, 7, 14, 21 and 28. After the 28-day growth phase, each loop was flushed from an initial flow of 0·4 l s^−1^ to a maximum flow of 4·5 l s^−1^ to assess the mechanical stability of biofilms.

### Water physico-chemical analysis

Water samples were collected on the day of coupon sampling during the growth phase and before and after flushing. The water samples were analysed for several physico-chemical factors (i.e. pH, temperature, chlorine, iron and manganese). Every analysis was performed in triplicate, and the average of the three replicates was calculated. Free chlorine was measured using a HACH DR/2010 spectrophotometer. Measurements of temperature and pH were made using a Hanna H1991003 meter and probes. Water samples for total iron and manganese were sent to an independent accredited laboratory AlControl Laboratories (Deeside, UK) for analysis. During flushing, turbidity was constantly measured by a Chemtrac TM2200 turbidity meter installed in the experimental facility to measure the particles mobilized as described in Sharpe *et al*. (2010).

### Sample collection for DNA extraction from bulk water and biofilms

To study changes in the bacterial community structure over time, PWG coupons were collected in triplicate from each loop during the material build-up phase at specific sampling days (days 0, 3, 7, 14, 21 and 28). Coupons were collected on day 0 after the disinfection process when chlorine concentrations matched that of the inlet water. Coupons collected on day 0 were used as controls. In total, 54 coupons were collected during the 28-day growth phase.

To assess the mechanical stability of the material developed on the pipe and the influence of flushing on bacterial community structure, coupons were also collected in triplicate from each loop before and after flushing the system (total of 18 coupons). To examine the impact that flushing had on the bulk water, triplicate 1l pre- and post flushing samples were taken directly from the outlet of each of the three loops. In total, 18 water samples were collected for this experiment and filtered through 0·22-*μ*m nitrocellulose membrane filters (Millipore UK Ltd., Watford, UK).

Biofilms were removed from PWG coupons, and cells were concentrated in nitrocellulose membrane filters as described in (Deines *et al.* 2010). Filters containing water and biofilms samples were kept in the dark and at −80°C for subsequent DNA analysis.

DNA extraction, including samples from day 0, was carried out by a method based on proteinase K digestion followed by a standard phenol/chloroform–isoamyl alcohol extraction (Neufeld *et al.* 2007). The quantity and purity of the extracted DNA were assessed using Nanodrop ND-1000 spectrophotometer (NanoDrop, Wilmington, DE).

### Terminal restriction fragment length polymorphism (T-RFLPs) analysis

A fragment of approximately 490 bp of the bacterial 16S rRNA gene, targeting the region V1-V3, was amplified using primer pair 63F (5′-CAGGCCTAACACATGCA AGTC-3′) and 518R (5′-CGTATTACCGCGGCTGCTCG-3′)(Girvan *et al.* 2003). The oligonucleotide 63F was labelled at the 5′ end with the phosphoramidite dye FAM (Applied Biosystems, Life Technologies Ltd, Paisley, UK). PCR mixtures were carried out using 12·5 *μ*l of TaqReadyMix with MgCl_2_ (Sigma-Aldrich Company Ltd., Dorset, UK) and 5 *μ*mol l^−1^ of each primer, using 1 *μ*l of template DNA, and sterile nuclease-free water to a final volume of 25 *μ*l. PCR conditions were 5 min at 95°C; 30 cycles of 30 s at 94°C, 30 s at 55°C and 1 min at 72°C; and a final extension for 10 min at 72°C. PCR products were purified using QIAquick PCR Purification Kit (Qiagen Inc., Valencia, CA) and then digested at 37°C for 2 h with restriction enzyme *Alu I* (Roche Diagnostics Ltd., Burgess Hill, UK). Desalted restriction digests were mixed with 1 *μ*l of deionized formamide and 0·5 *μ*l of a ROX-labelled Genescan 500-bp internal size standard (Applied Biosystems), denatured at 94°C for 3 min and immediately transferred to ice. T-RFLPs were separated by capillary electrophoresis using an automated genetic analyser ABI3730 (Applied Biosystems). Differences in abundance and length of T-RFLPs were determined by comparison with the known size internal standard, and the actual fragment sizes were estimated by interpolation using a Local Southern algorithm with the software GeneMapper 3.7 (Applied Biosystems). Terminal fragments smaller than 50 bp and with a peak height of <50 were excluded, considered background noise and eliminated from the analysis.

### Statistical analysis of T-RFLPs profiles

T-RFLPs were aligned on the basis of fragments length and peak areas using the T-Align Software (Smith *et al.* 2005). The aligned T-RFLPs were square root transformed, and Bray–Curtis similarity matrixes were calculated using the software primer v6 (PRIMER-E, Plymouth, UK). Bray–Curtis similarity matrixes were visualized using multiple dimensional scaling (MDS) diagrams. Analysis of similarity statistics (ANOSIM) was calculated using the same Bray–Curtis distance matrix to test the significance of differences among samples based on hydraulic regimes and flushing.

### Cloning and sequencing

Based on T-RFLPs results and to detect main shifts in biofilm bacterial composition, selected DNA samples obtained from biofilms grown under steady state conditions on days 7, 14 and 28 were PCR-amplified with bacterial primers 27F (5′-AGAGTTTGATCCTTGGCTCAG-3′) and 1492R (5′-GCYTACCTTGTTACGACTT-3′) (Lane 1991). PCR conditions were 5 min at 94°C, 30 cycles of 30 s at 94°C, 30 s at 54°C, and 90 s at 72°C and a final extension for 10 min at 72°C. The PCR master mix was prepared as explained in the previous section. PCR products from three biological replicates were pooled and purified using the QIAquick PCR Purification Kit (Qiagen Inc.). Purified PCR products were cloned using the pGEM-T Easy Vector Systems (Promega UK Ltd, Southampton, UK), and ligations were performed overnight at 4°C. Transformations were carried out using competent cells of *Escherichia coli* JM109, following manufacturer's instructions (Promega). Transformants were selected by ampicillin resistance, and blue-white screening was performed to identify clones with inserts (Sambrook *et al.* 1989). Inserts were sequenced from both directions using plasmid-vector-specific primers M13F (5′-CGCCAGGGTTTTCCCAGTCACGAC-3′) and M13R (5′-TAACAATTTCACACAGGA-3′) primers. Samples were purified with EXOSAP and sequenced at The University of Sheffield, Medical School with an Applied Biosystems 3730 automated DNA analyser.

### Sequencing and phylogenetic analysis

DNA was sequenced in both directions and consensus sequences obtained using the Phred-Phrap programs (Phil Green, Brent Ewing, University of Washington) by utilizing Perl scripts written by NBAF-Edinburgh and modified by Dr Gavin Horsburgh at NBAF-Sheffield, UK. Sequences were analysed for chimeric artefacts using DECIPHER (Wright *et al.* 2012), and any chimeric sequences identified were removed from the clone libraries for further analysis. The MultiClassifier tool based on naive Bayesian classifier in the Ribosomal Database Project (RDP) II (Cole *et al.* 2009) was used to classify query sequences at a confidence threshold of 80% (Wang *et al.* 2007). The online analysis function from the RDP was used to align sequences by the Infernal aligner (Nawrocki *et al.* 2009) and to cluster the aligned sequences by the complete-linkage clustering method; the cluster files were used to calculate Shannon diversity index (Shannon and Weaver 1963) and Chao richness estimator (Chao 1984). Column formatted distance matrices were generated using the RDP online analysis tool and used in mothur (Schloss *et al.* 2009) to calculate a Venn diagram with the number of shared OTUs between samples at 97% sequence similarity cut-off. The Basic Logic Alignment Search Tool (blast) (Altschul *et al.* 1990) was used to evaluate similarities with sequences deposited in the GenBank and to create a table with the summarized blast outputs at species level.

In this study, GenBank and the RDP provided similar taxonomic assignments from domain to genus. However, at species level, GenBank provides a measurement of the percent identity of a sequence and RDP measures relatedness values which is close to but inferior than the GenBank percent identity (Clarridge 2004). As a consequence, we have included the results from blast in Table2 to support the information obtained by the RDP.

Sequences included in the analysis were submitted to the GenBank and are available under the accession numbers KF611923-KF611976.

### Confocal laser scanning microscopy

Three replicate coupons with biofilm grown under steady state conditions were removed on days 7, 14 and 28. These particular coupons were selected to match the data obtained from cloning and sequencing the 16S rRNA gene. The flat insert section was separated from the coupon and fixed in 5% formaldehyde for 24 h, then transferred to phosphate buffer solution (PBS) and stored at 4°C until analysed. After fixing, the inserts were stained with 20 *μ*mol l^−1^ Syto 63 for 30 min. Syto 63 is a cell-permeative nucleic acid stain which is used to visualize cells (McSwain *et al.* 2005). Once stained, the samples were washed three times in sterile water and air-dried for 10 min before being stored in the dark at 4°C (<1 month). Imaging was performed at the Kroto Research Institute Confocal Imaging Facility, using a Zeiss LSM 510 Meta Confocal Florescent Microscope. Images were taken using an ×20 EC Plan Neoflaur Objective (0·5 NA) at 31·54 ų per pixel speed, pin hole set to an optical slice of 4·7 *μ*m, resolution 832 × 832 pixels and a frame size of 420 × 420 *μ*m. lsm510 Image Examiner Software was used to visualize the images (Zeiss, UK). Each insert was imaged for five random fields of view to provide an accurate representation of cell coverage. The images were then processed to extract a relative quantification of the biofilm at each layer. The images were firstly un-mixed, based on the spectral response of the Syto 63 at different laser wavelengths, to remove any influence of the plastic coupon substrate. The images were then median filtered to reduce noise, whereby the value of any pixel is calculated as the median of itself and the 8 pixels that surround it. Finally, the area of the biofilm per slice was calculated by the count of the pixels above a threshold, chosen to further reduce noise from the microscope and stain.

## Results

### Water physico-chemical analysis

**Figure 2:**
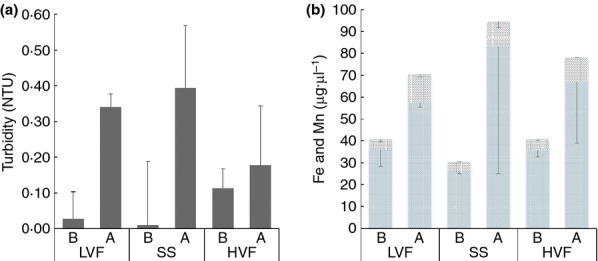
(a) Differences in bulk water turbidity (NTU) and (b) iron and manganese (*μ*g·*μ*l^−1^) concentrations among the three hydraulic regimes before and after flushing the system. B (before flushing) and A (after flushing). SS (steady state); LVF (low varied flow); HVF (high varied flow). Please note that for (a), the standard deviations are conventionally plotted positively to the bars. However, for (b), this is not possible due to the composite iron and manganese data being displayed. Figure (b) hence has standard deviation bars plotted negatively. (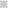) Fe; (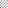) MN.

Table1 shows the characteristics of the water at specific sampling days during the 28 days of growth phase. pH values were near neutral (7·14–7·77) for most of the sampling days with the exception of day 21 with a slightly higher value of 8·28. The temperature of the facility was set to operate at 16°C, and during the experiment, water temperature fluctuated from 15·9 to 16·27°C. Average iron concentration of the water samples obtained at different sampling times during the 28-day accumulation phase test was 20·7 *μ*g l^−1^ ± 2·27 reaching a maximum on day 21 of 24 *μ*g l^−1^. Manganese concentrations were also highly consistent ranging from 2·1 to 2·9 *μ*g l^−1^. Free chlorine was high (0·44 mg l^−1^) on day 0 when the experiment started due to the filling of the system with fresh drinking water; the subsequent water samples once the system residence time of 24 h was established gave values ranging from 0·1 to 0·17 mg l^−1^. During the flushing experiment, free chlorine levels ranged between 0·19 and 0·28 mg l^−1^ (data not shown), showing higher values than those measured during the growth phase due to the refilling of the reservoir with fresh water between flushing successive loops. Figure2 shows the changes in levels of turbidity, iron and manganese concentration before and after flushing the system. Before flushing, the pipe loop which had been operated at the higher varied flow had higher turbidity levels (0·114 NTUs) than the other loops. After flushing the system, water turbidity levels and simultaneously iron and manganese concentrations increased. This was greatest in steady state and least in high varied flow, supporting previous laboratory investigations (Sharpe *et al.* 2010) and fieldwork observations (Husband *et al.* 2008) that daily hydraulics, characterized by daily peak, influences discolouration response.

**Table 1:** Average value (*n* = 3) and standard deviation of the physico-chemical parameters analysed in the bulk water samples during the 28 days of growth phase

Sampling day	pH	*T* (°C)	Fe (*μ*g l^−1^)	Mn (*μ*g l^−1^)	Free Chlorine (mg l^−1^)
0	7·77 ± 0·05	15·93 ± 0·11	22·0 ± 0·00	2·1 ± 0·00	0·44 ± 0·00
7	7·14 ± 0·00	16·20 ± 0·00	18·7 ± 0·58	2·1 ± 0·00	0·10 ± 0·02
14	7·43 ± 0·02	16·07 ± 0·05	19·7 ± 1·15	2·3 ± 0·11	*
21	8·28 ± 0·00	16·27 ± 0·11	24·0 ± 2·65	2·9 ± 0·70	0·11 ± 0·01
28	7·30 ± 0·10	16·03 ± 0·05	19·0 ± 0·00	2·9 ± 0·06	0·17 ± 0·03

* Below detection.

### Analysis of T-RFLPs profiles

#### Succession over time

**Figure 3:**
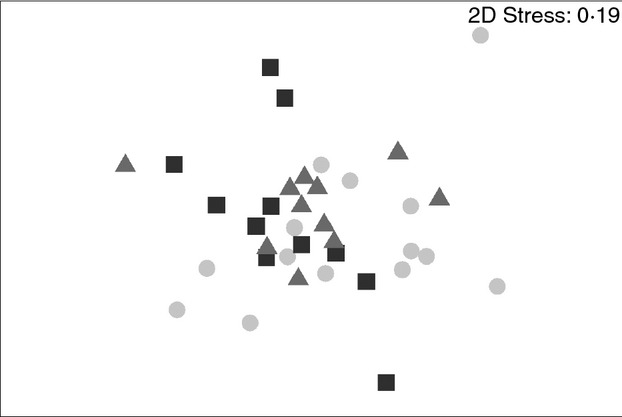
Nonmetric Multidimensional Scaling (MDS) ordination based on a Bray–Curtis resemblance matrix calculated from T-RFLPs profiles during biofilm growth phase. The plot shows distribution of samples according to hydraulic regime. SS (steady state); LVF (low varied flow); HVF (high varied flow). (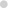) SS; (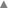) LVF; (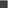) HVF

**Figure 4:**
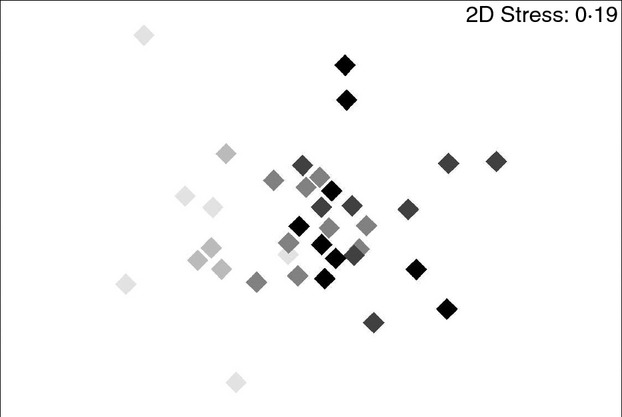
Two-dimensional plot of the nonmetric multidimensional scaling (MDS) ordination based on a Bray–Curtis resemblance matrix calculated from T-RFLPs profiles during biofilm growth phase. The plot shows differences in bacterial community structure at different days of biofilm development.(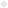) 3 days; (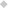) 7 days; (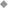) 14 days; (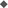) 21 days; (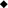) 28 days.

From the biofilm samples obtained on coupons collected on day 0 and used as controls, no DNA was obtained as confirmed both by Nanodrop quantifications and the lack of PCR amplifications from these samples. Similarly, not all the biofilm samples from day 3 and 7 yielded enough DNA to obtain good-quality amplicons for fingerprinting analysis and these were excluded from the analysis. The nonmetric multidimensional scaling analysis (MDS) of the T-RFLPs from biofilm samples (*n* = 36) obtained during the growth phase did not show a clear separation of the bacterial communities developed under the three different hydraulic regimes (Fig.3). However, when the factor analysed is time, changes in the bacterial community fingerprints of biofilm samples were detected (Fig.4). The analysis of similarity (ANOSIM) revealed that these temporal differences were particularly significant between days 7 and 21 (*R* = 0·607, *P* = 0·02).

#### Flushing

**Figure 5:**
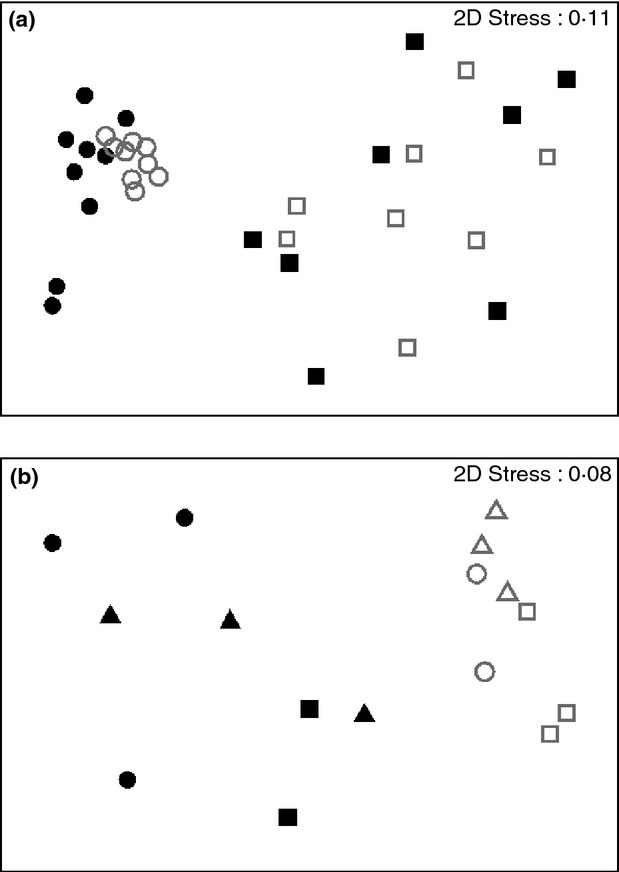
Two-dimensional plot of the nonmetric multidimensional scaling (MDS) ordination based on Bray–Curtis resemblance matrix calculated from T-RFLPs profiles during flushing (a) water *vs* biofilm and (b) pre- and post flushing. (a) (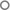) Water _B; (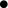) Water_A; (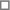) Biofilm _B; (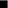) Biofilm_A (b) (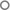) SS_B; (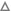) LVF_B; (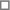) HVF_B; (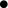) SS_A; (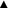) LVF_A; (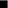) HVF_A

Flushing of the pipes was used to gain a practically relevant measure of biofilm stability and to assess the potential for detachment of material from the pipe walls into the bulk water. Terminal restriction fragments were obtained from 18 water samples and 16 biofilm samples during the flushing experiment and differences in the structure of the bacterial communities before and after flushing were analysed. Eighteen biofilm samples were collected during the flushing experiment; however, two samples (one before flushing at LVF and one after flushing at HVF) did not yield good amplifications for T-RFLPs analysis, and consequently, only 16 samples were used. The nonmetric MDS of the T-RFLPs profiles (Fig.5a) showed a clear separation between bacterial communities from the bulk water and those inhabiting the biofilms, ANOSIM (*R* = 0·852, statistic = 0·001). When considering only pre- and postflushing biofilm samples (*n* = 16) (Fig.5b), differences were detected between different hydraulic regimes (LVF *vs* HVF = 0·479, *P* = 0·06, SS *vs* HVF = 0·375, *P* = 0·13 and SS *vs* LVF = 0·143, *P* = 0·26) between pre- and postflushing samples (LVF = 0·77, *P* = 0·1, SS = 0·917, *P* = 0·1, HVF = 1, *P* = 0·1).

#### Analysis of sequencing data from days 7, 14 and 28 clone libraries

**Figure 6:**
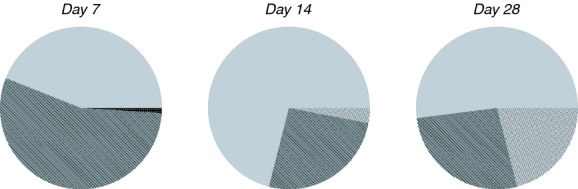
Pie charts showing the relative abundance of bacterial class in the three clone libraries from days 7, 14 and 28 at steady-state conditions. (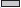) Betaproteobacteria; (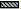) Gammaproteobacteria; (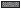) Flavobacteria; (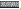) Alphaproteobacteria.

**Figure 7:**
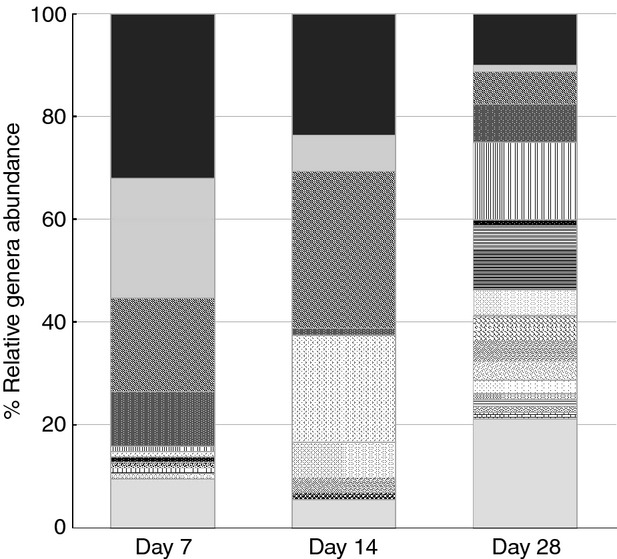
Relative abundance of bacterial genera found in the clone libraries according to the RDP classifier. 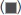 Pseudomonas; 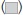 Janthinobacterium; 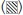 Methylophilus; 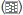 Stenotrophomonas; 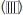 Undibacterium; 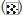 Dechloromonas; 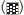 Acidovorax; 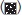 Bacteroidetes; 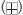 Flavobacterium; 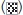 Curvibacter; 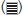 Porphyrobacter; 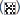 Sphingomonas; 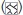 Nevskia; 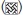 Sphingopyxis; 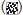 Novosphingobium; 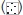 Methylobacterium; 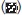 Acinetobacter; 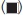 Unclassified_Methylophilaceae; 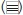 Unclassified_Rhizobiales; 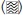 Unclassified_Erythrobacteraceae; 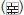 Unclassified_Pseudomonadaceae; 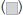 Unclassified.

As the T-RFLPs analysis did not show significant differences over time between different hydraulic regimes (Fig.3), we selected steady-state biofilm samples to assess changes in the bacterial community structure over time. With this aim, three clone libraries were constructed from biofilm samples developed on coupons under steady state conditions on days 7, 14 and 28. These days were selected because not enough amplicons were obtained from 3-day-old biofilms and changes were detected in the T-RFLPs profiles from these 3 days. After the removal of chimeric artefacts, a total of 237 sequences were obtained from the three libraries. The analysis of 16S rRNA gene sequences using the RDP II classifier showed that all three libraries were dominated by sequences related to the phylum *Proteobacteria* (Fig.6). The *Betaproteobacteria* subdivision was the predominant group in all the biofilm samples followed by *Gammaproteobacteria* and *Alphaproteobacteria* over the studied period of time. Over time, the percentage of *Betaproteobacteria* decreased and *Alphaproteobacteria* increased. Figure7 shows the results obtained at genera level for the sequences analysed using the RDP Naïve Bayesian classifier at a sequence threshold of 80%. Most of the clones selected on day 7 were closely related to the genus *Pseudomonas* (32%) followed by *Janthinobacterium* (23%), *Methylophilus* (18%) and *Stenotrophomonas* (11%). In the clone library obtained from day 14, *Methylophilus* was the most represented genus (30·5%) followed by *Pseudomonas* (23·6%), *Dechloromonas* (21%) and *Curvibacter* (7%). The percentage of clones related to *Pseudomonas* spp. decreased over time, and on day 28, they only represented 10% of the total number of clones in the library; other genera were more abundant in this particular clone library such as *Undibacteria* (15%) and *Porphyrobacter* (12·5%). However, on day 28, a high proportion of clones (21%) were not classified at genus level.

Table2 shows the results obtained from the GenBank database search at species level using the blast algorithm. The majority of *Gammaproteobacteria* in the clone library of day 7 were affiliated to the genus *Pseudomonas*. The remaining clones in this phylum were related to *Stenotrophomonas maltophilia*. Within the group of *Betaproteobacteria,* clones related to *Janthinobacterium* sp.*, Methylophilus methylotrophus* and *Duganella* sp. were highly represented*;* other clones were related to species of *Acidovorax* sp.*, Curvibacter gracilis and Dechloromonas* sp. With low sequence similarity (<95%), we detected one clone related to *Herbapirillum* sp.*,* another clone related to *Methylotenera mobilis* and one clone showed only 93% similarity to *Flavobacterium* sp. which belongs to the *Flavobacteria* phylum. On day 14, the presence of *Betaprotebacteria* representatives increased and species related to other genera such as *Methylomonas clara*, *Sphingopyxis* sp. and *Methylovorus glucosotrophus* were abundant; however, most of the other clones in these bacterial phyla were related to unknown uncultured bacteria. *Gammaproteobacteria* was mainly represented by species of *Pseudomonas* (21%) in particular *Pseudomonas fluorescens. Alphaproteobacteria* is represented in this clone library by only two clones related to *Sphingopyxis* sp. Finally, within the clone library of day 28, the *Betaproteobacteria* phylum was represented mainly by clones related to *Comamonadaceae bacterium* (97% similarity), *Undibacterium* sp. (similarity > 95%)*, Methylophylus methilotrophus* and *Oxalobacteriaceae bacterium*. Other *Betaproteobacteria* were represented by only one clone. Within *Gammaproteobacteria,* the majority of the clones were highly similar to *Stenotrophomonas maltophilia* (7·4% of clones) and different species of *Pseudomonas* spp. and *Nevskia* spp. In this clone library, the *Alphaproteobacteria* phylum was mainly represented by clones related to species such as *Porphyrobacter sanguineus* (13·4%), *Sphingomonas* sp. and several species of *Methylobacterium* sp. and *Novosphingobium* sp.

**Table 2:** Phylogenetic affiliations and percentage of similarity between the cloned 16S rRNA gene and its closest relative in the NCBI database using the blast algorithm

	Closest relative in GenBank	No. of clones	Similarity %	Accession number	Bacterial group
Clone Library Day 7	*Janthinobacterium* sp. *HC3-3*	19	97	JF312973.1	*Betaproteobacteria*
	*Pseudomonas* sp. *BcW159*	14	99	FJ889609.1	*Gammaproteobacteria*
	*Methylophilus methylothrophus*	13	98–99	AB193724.1	*Betaproteobacteria*
	*Pseudomonas* sp. *HC2-16*	9	99	JF312964.1	*Gammaproteobacteria*
	*Stenotrophomas maltophila R551-3*	8	99	NR_074875.1	*Gammaproteobacteria*
	*Duganella* sp. *S21012a*	4	97–98	AB495351.1	*Betaproteobacteria*
	*Pseudomonas fluorescence LMG14674*	4	99	GU198124.1	*Gammaproteobacteria*
	*Acidovorax* sp.*BSB421*	1	99	Y18617.1	*Betaproteobacteria*
	*Curvibacter gracilis*	1	99	AB109889.1	*Betaproteobacteria*
	*Dechloromonas* sp. *SIUL*	1	99	AF170356.1	*Betaproteobacteria*
	*Herbaspirillum* sp. *PIV.34*.*1*	1	95	AJ505863.1	*Betaproteobacteria*
	*Methylotenera mobilis*	1	94	AB698738.1	*Betaproteobacteria*
	*Flavobacterium* sp. *WB2*.*1-78*	1	93	AM167559.1	*Flavobacteria*
	*Pseudomonas* sp. *BXFJ-8*	1	99	EU013945.1	*Gammaproteobacteria*
	*Pseudomonas veronni*	1	99	AB334768.1	*Gammaproteobacteria*
Clone Library Day 14	*Pseudomonas fluorescens LMG 14674*	14	98–99	GU198124.1	*Gammaproteobacteria*
	*Dechloromonas hortensis MA-1*	13	98–99	NR_042819.1	*Betaproteobacteria*
	*Methylophilus rhizosphaerae strain 103a*	8	98–99	AB698737.1	*Betaproteobacteria*
	*Methylophilus methylothrophus*	7	99	AB193724.1	*Betaproteobacteria*
	*Curvibacter gracilis strain 7-1*	4	97–98	NR_028655.1	*Betaproteobacteria*
	*Methylomonas clara strain DSM 6330*	4	96–99	HF564897.1	*Betaproteobacteria*
	*Janthinobacterium* sp. *HC3-3*	2	96–99	JF312973.1	*Betaproteobacteria*
	*Janthinobacterium* sp. *SON-1402*	2	96–99	JX196629.1	*Betaproteobacteria*
	*Sphingopyxis* sp. *BZ3 0*	2	99	GQ131578.1	*Alphaproteobacteria*
	*Uncultured bacterium clone 1C226551*	2	96	EU799001.1	*Betaproteobacteria*
	*Uncultured Betaproteobacteria clone AEP-eGFP-*	2	99	FJ511736.1	*Betaproteobacteria*
	*Acinetobacter* sp. *K7SC-11A*	1	99	JF99965.1	*Gammaproteobacteria*
	*Dechloromonas* sp. *JD15*	1	100	JN873345.1	*Betaproteobacteria*
	*Dechloromonas* sp. *SIUL*	1	98	AF170356.1	*Betaproteobacteria*
	*Methylovorus glucosotrophus strain DSM68 74T*	1	95	FR733702.1	*Betaproteobacteria*
	*Pseudomonas* sp. *EC3(2012)*	1	99	JX912405.1	*Gammaproteobacteria*
	*Stenotrophomonas* sp. *Bg23-2*	1	99	HF548414.1	*Gammaproteobacteria*
	*Uncultured bacterium clone 2M04*	1	99	EU835430.1	*Betaproteobacteria*
	*Uncultured bacterium clone EDWO7B003-11*	1	96	HM066437.1	*Betaproteobacteria*
	*Uncultured bacterium cloneMB50-16*	1	97	JN825265.1	*Betaproteobacteria*
	*Uncultured Methylobacillus clone R15-96*	1	95	JF808844.1	*Betaproteobacteria*
Clone Library Day 28	*Comamonadaceae bacterium ED16*	23	97	FJ755906.1	*Betaproteobacteria*
	*Porphyrobacter sanguineus*	11	99	AB062106.1	*Alphaproteobacteria*
	*Stenotrophomonas maltophilia R551-3*	6	99	NR_074875.1	*Gammaproteobacteria*
	*Undibacterium* sp. *C3*.	6	96	JQ417431.1	*Betaproteobacteria*
	*Methylophilus methylothrophus*	4	99	AB193724.1	*Betaproteobacteria*
	*Oxalobacteraceae bacterium CHNTR40*	4	99	DQ337591.1	*Betaproteobacteria*
	*Pseudomonas* sp. *BFXJ-8*	3	99	EU013945.1	*Gammaproteobacteria*
	*Sphingomonas* sp. *M16*	3	99	GU086440.1	*Alphaproteobacteria*
	*Undibacterium* sp. *M4-14*	3	97	HE616176.1	*Betaproteobacteria*
	*Pseudomonas veronii*	2	99	AB334768.1	*Gammaproteobacteria*
	*Uncultured Nevskia* sp. *Clone T13M-B4*	2	98	JN860401.1	*Gammaproteobacteria*
	*Acidovorax* sp. *isolate G8B1*	1	99	AJ012071.1	*Betaproteobacteria*
	*Janthinobacterium* sp. *HC3-3*	1	99	JF312973.1	*Betaproteobacteria*
	*Janthinobacterium* sp. *S21124*	1	99	D84576.2	*Betaproteobacteria*
	*Methylobacterium marchantiae*	1	99	AB698714.1	*Alphaproteobacteria*
	*Methylobacterium* sp. *Mp3*	1	99	EF015480.1	*Alphaproteobacteria*
	*Nevskia ramosa*	1	98	AJ001343.1	*Gammaproteobacteria*
	*Nevskia* sp. *KNF011*	1	99	AB426555.1	*Gammaproteobacteria*
	*Novosphingobium* sp. *FND-3*	1	99	DQ831000.1	*Alphaproteobacteria*
	*Novosphingobium* sp. *HLT3-9*	1	99	JX9493761.1	*Alphaproteobacteria*
	*Pseudomonas fluorescens*	1	98	AM410631.1	*Gammaproteobacteria*
	*Pseudomonas putida*	1	99	EU275363.1	*Gammaproteobacteria*
	*Pseudomonas* sp. *HC2-30*	1	96	JF312926.1	*Gammaproteobacteria*
	*Sphingomonas sechinoides S32312*	1	99	AB649019.1	*Alphaproteobacteria*
	*Uncultured Methylophilaceae bacterium*	1	98	HE648207.1	*Betaproteobacteria*
	*Undibacterium parvum*	1	98	AM397629.1	*Betaproteobacteria*

**Figure 8:**
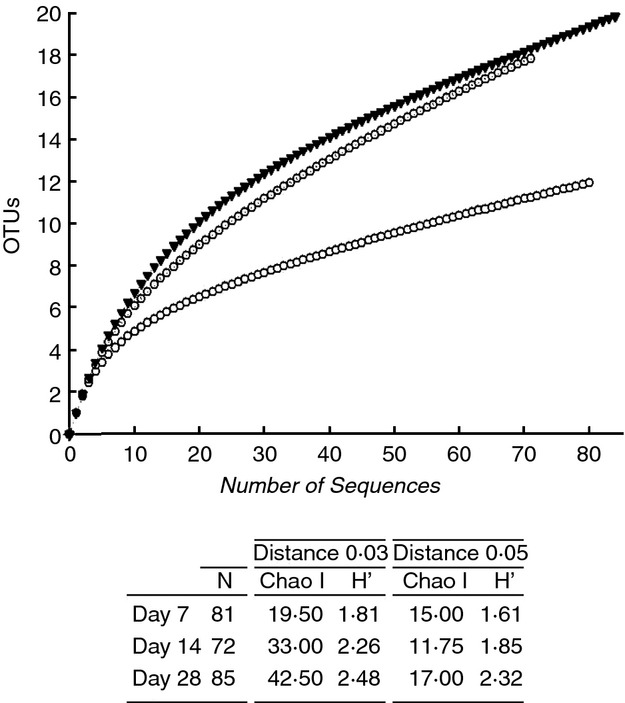
Rarefaction curves at 97% of sequence similarity showing observed OTUS for the three clone libraries (samples from days 7, 14 and 28). (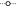) Day 7; (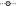) Day 14; (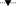) Day 28.

**Figure 9:**
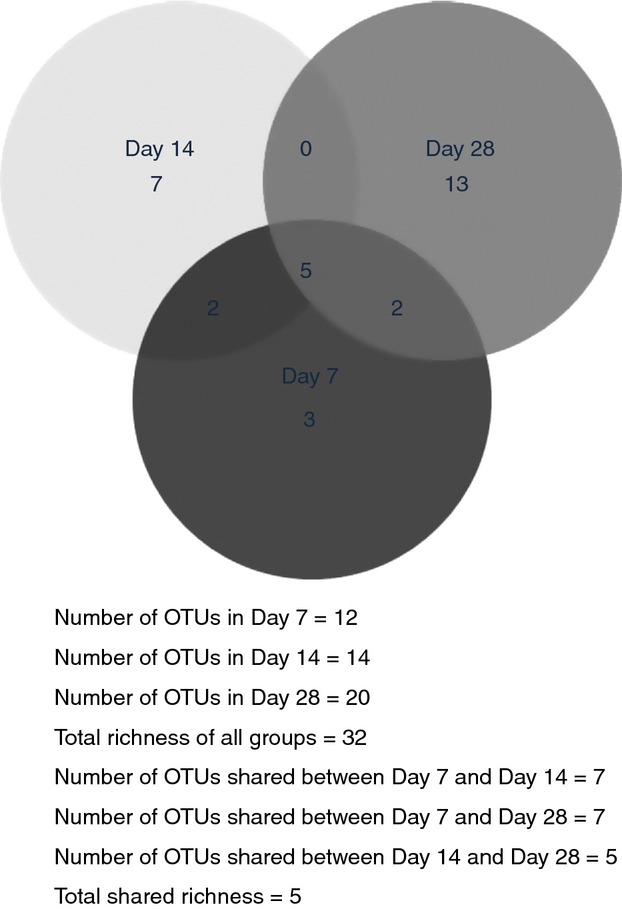
Venn diagram of bacterial OTUs clustered with a 3% distance similarity threshold, showing the number of OTUs shared by the clone libraries from days 7, 14 and 28. Number of OTUs in day 7 = 12; number of OTUs in day 14 = 14; number of OTUs in day 28 = 20; total richness of all groups = 32; number of OTUs shared between day 7 and day 14 = 7; number of OTUs shared between day 7 and day 28 = 7; number of OTUs shared between day 14 and day 28 = 5; total shared richness = 5.

Aligned sequences were clustered into OTUs at 95% (used as genus level) and 97% (used as species level) sequence similarities thresholds, and rarefaction curves were plotted for the observed OTUs at 97% cut-off (Fig.8). As it was expected, the rarefaction curves showing the number of OTUs per clone library did not show a plateau due to the limited number of clones in the sequenced libraries. Species richness (Chao I) and diversity (Shannon index) of the clone libraries calculated at 95 and 97% cut-offs increased over time from day 7 to 28 with the exception of Chao I at 95% cut-off for day 14 (Fig.8). The Venn diagram generated to calculate the shared OTUs between clone libraries showed that the total estimated richness of all groups at 97% sequence similarity threshold (i.e. species level) was 32 OTUs and that the estimated richness shared by the three clone libraries was 5 OTUs (Fig.9). The data indicate that after the initial first week of material development within the pipes, the bacterial community changes towards a more diverse community.

#### Microscopy analysis

**Figure 10:**
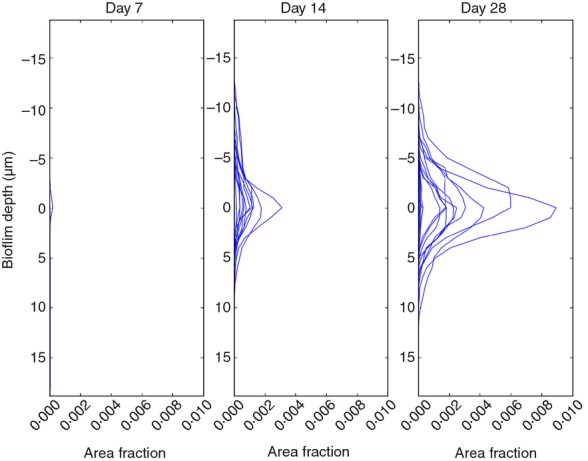
Analysis of confocal images showing changes in cell coverage over time. The graphs represent the biofilm relative area fraction of five fields of view of three plastic inserts obtained from PWG coupons. Fields of view below the fluorescence threshold are not represented in the graph.

Given that no significant changes in the bacterial community structure were detected using fingerprinting techniques between the three different hydraulic regimes, only biofilms developed under steady state conditions were imaged to assess the rate of biofilm build-up in our system. Graphs in Fig.10 represent changes in biofilm area fraction, calculated to quantify changes in cell coverage (i.e. the relative area of each 2D image covered by stained cells) on pipe surfaces over time under steady state conditions. On day 7, most of the analysed field of views from the three different coupons were not above the fluorescence threshold established to distinguish microbial cells from fluorescence emitted by the plastic coupon and considered as background or noise. Throughout time, cell coverage increased from a maximum area fraction on day 14 of 0·003 to a maximum fraction on day 28 of 0·009, showing that cell coverage can be triplicated after 2 weeks of biofilm development under steady state conditions.

## Discussion

### Bacterial community composition and succession over time

The T-RFLPs profiles (Fig.4) and the sequencing analysis of the clone libraries (Figs6–9) showed clear changes in the bacterial community composition over time. The dominant phylum in the clone libraries was *Proteobacteria,* and within this, *Gammaproteobacteria* and *Betaproteobacteria* were predominant (Fig.6). However, the percentage of these two bacterial classes varied over time and representatives of the *Alphaproteobacteria* increased in the day 28 clone library. *Gammaproteobacteria*, *Betaproteobacteria* and *Alphaproteobacteria* were also the predominant phyla in a previous study, where the bacterial composition of biofilms obtained from the same experimental distribution system at day 28 was analysed using 454 pyrosequencing (Douterelo *et al.* 2013). The dominance of *Proteobacteria* in biofilms has been previously observed in other drinking water ecosystems, for example in rubber coated valves of a nonchlorinated network (Schmeisser *et al.* 2003), in a nonchlorinated model system made of stainless steel and supplied with groundwater (Martiny *et al.* 2005), in a simulated drinking water supply system using two different materials steel and polymethylmetacrylate (Lopes *et al.* 2009) and in a pilot distribution system made of cement-lined cast iron (Mathieu *et al.* 2009). This study corroborates the predominance of this bacterial group for biofilms up to 28 days old in DWDS made of HDPE, a representative material used in distributions systems worldwide, supplied with surface water and with a chlorine disinfectant residual.

Additional OTU-based analysis of the clone libraries confirmed a gradual increase in species richness and diversity over time during the 28 days of material accumulation (Fig.8). In agreement with our results, previous research on biofilms showed that only a limited number of pioneer micro-organisms are able to initially attach to a surface, change the characteristics of the surface and start to form biofilms. However, several of these studies were carried out on glass slides (Jackson *et al.* 2001; Paris *et al.* 2007; Doğruöz *et al.* 2009), which do not reflect realistic conditions in DWDS, or in a model DWDS made of stainless steel supplied with groundwater and without disinfectant residual (Martiny *et al.* 2003). We have confirmed that the process of bacterial biofilm succession occurs under realistic conditions in chlorinated drinking water networks, using a full-scale HDPE test loop facility, and we were also able to identify the bacteria involved in the initial development of biofilms in this type of DWDS.

We also observed that bacterial community fingerprints were clearly different between bulk water and biofilms in pre- and postflushing samples (Fig.5a), suggesting that these two habitats share a limited number of bacteria and that only some free-living bacteria transported from the bulk water are able to attach to the pipe surfaces and form biofilms, supporting previous observations (Douterelo *et al.* 2013).

From the sequencing data, we can deduce that bacteria related to *Pseudomonas* spp., *Janthinobacterium* spp. and *Methylophilus* spp. were involved in the initial process of attachment to the pipe surface (Fig.7). The occurrence of these bacteria in the initial phase of biofilm formation can be explained due to their enhanced capability, when compared with other planktonic bacteria, of producing EPS when they establish contact with a surface and/or molecules required for cell-to-cell communication (Donlan 2002; Huq *et al.* 2008). *Pseudomonas* spp. is one of the most abundant bacteria found in water distribution systems (Martiny *et al.* 2005), and several species of *Pseudomonas*, like *Ps. fluorescens,* are capable of synthesizing surfactants involved in cell-to-cell communication (Simoes *et al.* 2003); others like *Ps. aeruginosa* can produce alginate and exopolysaccharides when in contact with different surfaces (Huq *et al.* 2008). Martiny *et al.* 2003, when studying long-term succession of biofilms in a model DWDS supplied with groundwater and using turbulent flow conditions (flow velocity of 0·07 m s^−1^), also found that bacteria related to *Pseudomonas* spp. were predominant in the initial attachment of cells to stainless steel. As a consequence, we can conclude that *Pseudomonas* spp. is an ubiquitous bacteria involved in the process of biofilm formation in DWDS made of different materials and under diverse hydraulic conditions.

We observed that after the initial colonization of a few bacterial species (from day 0 to 7), the modified surface of the pipe was attractive for other species (Table2). After 2 weeks of biofilm development, clones related to *Dechloromonas* spp. become highly abundant. *Pseudomonas* spp. losses its predominance, and new clones related to other species such as *Curvibacter*, spp. *Methylomonas* spp. and *Sphingopyxis* spp. were now present. Finally on day 28, higher OTUs richness and diversity were detected (Table2), and members of genera such as *Undibacterium* spp., *Porphyrobacter* spp., *Nevskia* spp. and *Sphingomonas* spp. became represented in the clone library (Fig.7). It has been hypothesized that these secondary colonizers are able to attach to a growing biofilm aided by their capability to use the metabolites excreted or the remains generated by the first colonizers (Szewzyk *et al.* 2000). In agreement with the DNA-based results, the analysis of confocal microscopy z-stack images obtained from steady state conditions showed that on day 7 only a limited number of cells attached to the coupon surface, making their detection difficult. Cell coverage increased substantially after another week of development in the system, and from day 14 to 28, the area covered by the biofilm increased up to three times.

Some of the bacterial genera detected in our clone libraries have been previously found in other drinking water-related ecosystems; for example, *Porphyrobacter* spp.*, Sphingopyxis* spp. and *Undibacterium* spp. were detected in biofilms of a membrane filtration system in a treatment plant (Kwon *et al.* 2011). Revetta *et al.* 2010, using RNA to develop 16S rRNA-based clone libraries, showed that metabolically active *Proteobacteria* such as *Porphyrobacter* spp. and *Sphingomonas* spp. were present in bulk water samples from a chlorinated drinking water network. However, from most of these studies, it was not clear at which stage in the process of biofilm formation these planktonic bacteria were able to attach to the pipe surface and/or to attach to a growing biofilm. From the data obtained in this study, we can conclude that species related to *Pseudomonas* spp., *Janthinobacterium* spp. *and Methylophilus* spp. were primary colonizers of the HDPE pipe surface and that other species such as *Dechloromonas* spp., *Curvibacter* spp. and *Sphingopyxis* spp. were probably secondary colonizers.

The role of most of the bacteria detected in drinking water biofilms remains uncertain. For example, *Dechloromonas* spp.*,* which was predominant in the clone library of day 14, are widely distributed in the environment (Vigliotta *et al.* 2010) and are known perchlorate-reducing bacteria (Oosterkamp *et al.* 2011), but their metabolic or physiological function in drinking water biofilms is not well known. Chlorate-reducing bacteria have been detected in DWDS in the USA, associated with releases of ammonium perchlorate by military- and aerospace-related industries (Urbansky 2002). Their presence in our chlorinated system might be related to their capability of reducing nitrate instead of chlorate, but this assumption needs further investigation (Oosterkamp *et al.* 2011). Similarly, *Methylophylus* spp.*,* which was highly abundant in the three clone libraries of this study and also in water meters from a DWDS (Hong *et al.* 2010), can oxidize methyl compounds and can possible carry out denitrification, but the particular function that makes them so abundant in the early stages of biofilm formation has not been clarified yet.

We were able to identify bacterial groups involved in initial attachment to HDPE pipes and subsequent adhesion of cells to growing biofilms in chlorinated drinking water networks, but further study into their specific functions and their role within biofilms is still necessary to move forward in this research field and hence to deliver strategies to manage and control biofilm development in DWDS. In addition, longer studies would be needed to evaluate the succession of bacterial communities in more mature biofilms and to establish if at some point the biofilm reaches a dynamic stability or equilibrium.

### Influence of hydraulic regimes on biofilm development and mechanical stability

Based on fingerprinting analysis (T-RFLPs), the hydraulic regimes did not affect the bacterial community structure during the first 28 days of biofilm development (Fig.3). This suggests that independently of the hydraulic regime, similar planktonic species were involved in the process of surface attachment and initial biofilm formation. Nevertheless, these results need to be interpreted with caution due to the impossibility of obtaining enough DNA for further 16S rRNA amplifications from all the samples on days 3 and 7 under varied flow conditions and the fact that fingerprinting techniques only yield information about the most abundant members of microbial communities. Another factor which might influence our results is that we have used a common reservoir to feed the three loops; as a consequence, the similar physico-chemical and microbiological characteristics of the water circulating within the system might contribute to the lack of influence of the hydraulic regimes in our experimental system.

By flushing, we removed attached micro-organisms from the pipes together with particles such as iron and manganese which are most likely confined within biofilms, as can be seen from turbidity and metal concentration changes after flushing (Fig.2). Combining the T-RFLPs results from pre- and postflushing biofilm samples (Fig.5) and the water turbidity data (Fig.2) obtained during flushing, it can be suggested that the hydraulic regimes did affect the mechanical stability of biofilms. Flushing removed different amounts of material from the pipes depending on the flow regime applied during the biofilm growth phase. Furthermore, the differences in T-RFLPs profiles found between pre- and postflushing samples (Fig.5) showed that bacterial community structure changed after flushing, suggesting that some members of the community were effectively removed by flushing, but other bacteria remained adhered to the pipe surfaces. We have also detected a shift in the bacterial community structure after flushing when using pyrosequencing to study in detail the role of bacteria in the process of water discolouration (Douterelo *et al.* 2013). We can infer from this study that hydraulic regimes did not significantly affect the bacteriological composition of the biofilms at the early stages of their formation but that they might influence the physical structure (e.g. shape and distribution on pipes) of the biofilm and potentially also their biochemical composition (i.e. amount of polysaccharides, lipids, etc.). Several biofilm-related studies showed that high shear stress and turbulent flow conditions can promote the development of more compact biofilms due mainly to the production of extracellular polymers; this trend was observed for example in a biofilm airlift suspension (Kwok *et al.*, 1998), using bench-scale biofilm-monitoring reactors (Manuel *et al.*, 2010) and in single-species biofilms consisting of *Ps. fluorescens* (Pereira *et al.*, 2002). The observed influence that different hydraulic regimes had on the cohesiveness of biofilms developed in our full-scale DWDS agrees with observations made in a rotary disk reactor by Abe *et al.*, (2012) that a more cohesive biofilm structure can be more resistant to external shear stress and detachment.

The enhanced capability for producing EPS in particular polysaccharides and/or adaptative physical structures like extracellular appendages in response to fluctuating flows might contribute to the mechanical stability of biofilms and might aid certain types of bacteria in remaining attached to a surface after an increase in flow velocity (Simoes *et al.* 2010). Accordingly, Liu *et al.*, 2002 observed that micro-organisms respond to shear forces by producing stronger biofilms under higher hydrodynamic shear forces and biofilms become more porous and weaker under low shear forces. In addition, it has been proven that quorum-sensing mechanisms are involved in the attachment and detachment of microbial cells from biofilms (Donlan 2002) and these mechanisms of communication might be enhanced under variable flow conditions. This study highlights that under representative hydraulic conditions, the strength of biofilm attachment to pipe surfaces is higher under varied flow conditions which can be translated into lower risk of water discolouration.

Preventing the attachment of specific primary colonizing bacteria to a pipe inner surface might be preferable than trying to eliminate micro-organisms within the EPS matrix of a mature biofilm. Effective control of biofilms in DWDS might require the design of specific antimicrobial agents or treatments to limit the presence of initial colonizing bacteria at the treatment plant and to for example inhibit their capabilities of initial attachment, EPS synthesis and/or cell-to-cell communication.

The present work provides information about the bacterial composition on the initial steps of biofilm formation in a chlorinated system made of HDPE pipes and about the mechanical stability of biofilms under different hydrological conditions which will contribute to better understand how biofilms form in DWDS and to improve current control strategies.

## References

[b1000] Abe Y, Skali-Lami S, Block JC, Francius G (2012). Cohesiveness and hydrodynamic properties of young drinking water biofilms. Water Res.

[b1] Altschul SF, Gish W, Miller W, Myers EW, Lipman DJ (1990). Basic local alignment search tool. J Mol Biol.

[b2] Andrews JS, Rolfe SA, Huang WE, Scholes JD, Banwart SA (2010). Biofilm formation in environmental bacteria is influenced by different macromolecules depending on genus and species. Environ Microbiol.

[b3] Batté M, Appenzeller BMR, Grandjean D, Fass S, Gauthier V, Jorand F, Mathieu L, Boualam M (2003). Biofilms in drinking water distribution systems. Rev Environ Sci Biotechnol.

[b4] Chao A (1984). Non parametric estimation of the number of classes in a population. Scand Stat Theory Appl.

[b5] Clarridge JE (2004). Impact of 16S rRNA gene sequence analysis for identification of bacteria on clinical microbiology and infectious diseases. Clin Microbiol Rev.

[b6] Cole JR, Wang Q, Cardenas E, Fish J, Chai B, Farris RJ, Kulam-Syed-Mohideen AS, McGarrell DM (2009). The Ribosomal Database Project: improved alignments and new tools for rRNA analysis. Nucleic Acids Res.

[b7] Costerton JW, Cheng KJ, Geesey GG, Ladd TI, Nickel JC, Dasgupta M, Marrie TJ (1987). Bacterial biofilms in nature and disease. Annu Rev Microbiol.

[b8] Deines P, Sekar R, Husband PS, Boxall JB, Osborn AM, Biggs CA (2010). A new coupon design for simultaneous analysis of in situ microbial biofilm formation and community structure in drinking water distribution systems. Appl Microbiol Biotechnol.

[b9] Doğruöz N, Göksay D, Ilhan-Sungur E, Cotuk A (2009). Pioneer colonizer microorganisms in biofilm formation on galvanized steel in a simulated recirculating cooling-water system. J Basic Microbiol.

[b10] Donlan RM (2002). Biofilms: microbial life on surfaces. Emerg Infect Dis.

[b11] Douterelo I, Sharpe RL, Boxall JB (2013). Influence of hydraulic regimes on bacterial community structure and composition in an experimental drinking water distribution system. Water Res.

[b12] Flemming H-C, Wingender J (2010). The biofilm matrix. Nat Rev Microbiol.

[b13] Flemming HC, Percival SL, Walker JT (2002). Contamination potential of biofilms in water distribution systems. Water Supply.

[b14] Ginige MP, Wylie J, Plumb J (2011). Influence of biofilms on iron and manganese deposition in drinking water distribution systems. Biofouling.

[b15] Girvan MS, Bullimore J, Pretty JN, Osborn AM, Ball AS (2003). Soil type is the primary determinant of the composition of the total and active bacterial communities in arable soils. Appl Environ Microbiol.

[b16] Hong P-Y, Hwang C, Ling F, Andersen GL, LeChevallier MW, Liu W-T (2010). Pyrosequencing analysis of bacterial biofilm communities in water meters of a drinking water distribution system. Appl Environ Microbiol.

[b17] Huq A, Whitehouse CA, Grim CJ, Alam M, Colwell RR (2008). Biofilms in water, its role and impact in human disease transmission. Curr Opin Biotechnol.

[b18] Husband PS, Boxall JB, Saul AJ (2008). Laboratory studies investigating the processes leading to discolouration in water distribution networks. Water Res.

[b19] Jackson CR, Churchill PF, Roden EE (2001). Successional changes in bacterial assemblage structures during epilithic biofilm development. Ecology.

[b1002] Kwok WK, Picioreanu C, Ong SL, van Loosdrecht MC, Ng WJ, Heijnen JJ (1998). Influence of biomass production and detachment forces on biofilm structures in a biofilm airlift suspension reactor. Biotechnol Bioeng.

[b20] Kwon S, Moon E, Kim TS, Hong S, Park HD (2011). Pyrosequencing demonstrated complex microbial communities in a membrane filtration system for a drinking water treatment plant. Microbes Environ.

[b21] Lane DJ, Stackebrandt E, Goodfellow M (1991). 16S/23S rRNA sequencing. Nucleic acid techniques in bacterial systematics.

[b22] Lee JW, Nam JH, Kim YH, Lee KH, Lee DH (2008). Bacterial communities in the initial stage of marine biofilm formation on artificial surfaces. J Microbiol.

[b23] Li X, Upadhyaya G, Yuen W, Brown J, Morgenroth E, Raskin L (2010). Changes in the structure and function of microbial communities in drinking water treatment bioreactors upon addition of phosphorus. Appl Environ Microbiol.

[b1003] Liu Y, Tay JH (2002). The essential role of hydrodynamic shear force in the formation of biofilm and granular sludge. Water Res.

[b24] Liu Y, Yang S-F, Li Y, Xu H, Qin L, Tay J-H (2004). The influence of cell and substratum surface hydrophobicities on microbial attachment. J Biotechnol.

[b25] Lopes FA, Morin P, Oliveira R, Melo LF (2009). Impact of biofilms in simulated drinking water and urban heat supply systems. Int J Environ Eng.

[b1005] Manuel CM, Nunes OC, Melo LF (2010). Unsteady state flow and stagnation in distribution systems affect the biological stability of drinking water. Biofouling.

[b26] Martiny AC, Jorgensen TM, Albrechtsen HJ, Arvin E, Molin S (2003). Long-term succession of structure and diversity of a biofilm formed in a model drinking water distribution system. Appl Environ Microbiol.

[b27] Martiny AC, Albrechtsen HJ, Arvin E, Molin S (2005). Identification of bacteria in biofilm and bulk water samples from a nonchlorinated model drinking water distribution system: detection of a large nitrite-oxidizing population associated with *Nitrospira* spp. Appl Environ Microbiol.

[b28] Mathieu L, Bouteleux C, Fass S, Angel E, Block JC (2009). Reversible shift in the alpha-, beta- and gamma-proteobacteria populations of drinking water biofilms during discontinuous chlorination. Water Res.

[b29] McSwain BS, Irvine RL, Hausner M, Wilderer PA (2005). Composition and distribution of extracellular polymeric substances in aerobic flocs and granular sludge. Appl Environ Microbiol.

[b30] Moritz MM, Flemming HC, Wingender J (2010). Integration of *Pseudomonas aeruginosa* and *Legionella pneumophila* in drinking water biofilms grown on domestic plumbing materials. Int J Hyg Environ Health.

[b31] Morvay AA, Decun M, Scurtu M, Sala C, Morar A, Sarandan M (2011). Biofilm formation on materials commonly used in household drinking water systems. Water Sci Technol: Water Supply.

[b32] Nawrocki EP, Kolbe DL, Eddy SR (2009). Infernal 1.0: inference of RNA alignments. Bioinformatics.

[b33] Neufeld JD, Vohra J, Dumont MG, Lueders T, Manefield M, Friedrich MW, Murrell JC (2007). DNA stable-isotope probing. Nat Protoc.

[b34] Oosterkamp MJ, Mehboob F, Schraa G, Plugge CM, Stams AJM (2011). Nitrate and (per)chlorate reduction pathways in (per)chlorate-reducing bacteria. Biochem Soc Trans.

[b35] Paris T, Skali-Lami S, Block J-C (2007). Effect of wall shear rate on biofilm deposition and grazing in drinking water flow chambers. Biotechnol Bioeng.

[b1006] Pereira MO, Kuehn M, Wuertz S, Neu T, Melo LF (2002). Effect of flow regime on the architecture of a *Pseudomonas fluorescens* biofilm. Biotechnol Bioeng.

[b36] Revetta RP, Pemberton A, Lamendella R, Iker B, Santo Domingo JW (2010). Identification of bacterial populations in drinking water using 16S rRNA-based sequence analyses. Water Res.

[b38] Sambrook J, Fritsch EF, Maniatis T (1989). Molecular Cloning: A laboratory Manual.

[b39] Schloss PD, Westcott SL, Ryabin T, Hall JR, Hartmann M, Hollister EB, Lesniewski RA, Oakley BB (2009). Introducing mothur: open-source, platform-independent, community-supported software for describing and comparing microbial communities. Appl Environ Microbiol.

[b40] Schmeisser C, Stockigt C, Raasch C, Wingender J, Timmis KN, Wenderoth DF, Flemming HC, Liesegang H (2003). Metagenome survey of biofilms in drinking-water networks. Appl Environ Microbiol.

[b41] Shannon CE, Weaver W (1963). The Mathematical Theory of Communication.

[b42] Sharpe RL, Smith CJ, Biggs CA, Boxall JB (2010). Pilot scale laboratory investigation into the impact of steady state conditioning flow on potable water discolouration.

[b43] Simoes M, Carvalho H, Pereira MO, Vieira MJ (2003). Studies on the behaviour of *Pseudomonas fluorescens* biofilms after ortho-phthalaldehyde treatment. Biofouling.

[b44] Simoes LC, Simoes M, Oliveira R, Vieira MJ (2007a). Potential of the adhesion of bacteria isolated from drinking water to materials. J Basic Microbiol.

[b45] Simoes LC, Simoes M, Vieira MJ (2007b). Biofilm interactions between distinct bacterial genera isolated from drinking water. Appl Environ Microbiol.

[b46] Simoes LC, Simoes M, Vieira MJ (2010). Influence of the diversity of bacterial isolates from drinking water on resistance of biofilms to disinfection. Appl Environ Microbiol.

[b47] Smith CJ, Danilowicz BS, Clear AK, Costello FJ, Wilson B, Meijer WG (2005). T-Align, a web-based tool for comparison of multiple terminal restriction fragment length polymorphism profiles. FEMS Microbiol Ecol.

[b48] Stoodley P, Sauer K, Davies DG, Costerton JW (2002). Biofilms as complex differentiated communities. Annu Rev Microbiol.

[b49] Szewzyk U, Szewzyk R, Manz W, Schleifer KH (2000). Microbiological safety of drinking water. Annu Rev Microbiol.

[b50] Urbansky ET (2002). Perchlorate as an environmental contaminant. Environ Sci Pollut Res Int.

[b51] Vigliotta G, Motta O, Guarino F, Iannece P, Proto A (2010). Assessment of perchlorate-reducing bacteria in a highly polluted river. Int J Hyg Environ Health.

[b52] Wang Q, Garrity GM, Tiedje JM, Cole JR (2007). Naïve Bayesian classifier for rapid assignment of rRNA sequences into the new bacterial taxonomy. Appl Environ Microbiol.

[b53] Wright ES, Yilmaz LS, Noguera DR (2012). DECIPHER, a search-based approach to chimera identification for 16S rRNA sequences. Appl Environ Microbiol.

[b54] Yu J, Kim D, Lee T (2010). Microbial diversity in biofilms on water distribution pipes of different materials. Water Sci Technol.

[b55] Zacheus OM, Iivanainen EK, Nissinen TK, Lehtola MJ, Martikainen PJ (2000). Bacterial biofilm formation on polyvinyl chloride, polyethylene and stainless steel exposed to ozonated water. Water Res.

